# The *Lutzomyia longipalpis* complex: a brief natural history of aggregation-sex pheromone communication

**DOI:** 10.1186/s13071-016-1866-x

**Published:** 2016-11-14

**Authors:** Carolina N. Spiegel, Denise B. dos Santos Dias, Alejandra S. Araki, James G. C. Hamilton, Reginaldo P. Brazil, Théresa M. Jones

**Affiliations:** 1 Departamento de Biologia Celular e Molecular, Instituto de Biologia, Universidade Federal Fluminense, Outeiro de São João Batista s/n, Valonguinho, Centro, Niterói, 24.020-150 RJ Brazil; 2 Departamento de Bioquímica, Instituto de Biologia Roberto Alcântara Gomes, Universidade do Estado do Rio de Janeiro, Rio de Janeiro, CEP: 20.551-030 RJ Brazil; 3 Laboratório de Biologia Molecular de Insetos, Instituto Oswaldo Cruz, FIOCRUZ, Rio de Janeiro, 21040-360 RJ Brazil; 4 Division of Biomedical and Life Sciences, School of Health and Medicine, Lancaster University, Lancaster, UK; 5 Laboratório de Doenças Parasitárias, Instituto Oswaldo Cruz, FIOCRUZ, Rio de Janeiro, 21040-900 RJ Brazil; 6 School of BioSciences, University of Melbourne, Melbourne, 3010 Australia

**Keywords:** *Lutzomyia longipalpis*, Sex pheromone, Aggregation pheromone, Species complex, Evolution, Sand flies

## Abstract

In this paper we review the natural history of pheromone communication and the current diversity of aggregation-sex pheromones in the sand fly *Lutzomyia longipalpis.* This species complex is the main vector of *Leishmania infantum,* the agent of visceral leishmaniasis in the Americas. The identification of variation in pheromone chemotypes combined with molecular and sound analyses have all contributed to our understanding of the extent of divergence among cryptic members of this complex. The importance of chemical signals as pre-mating barriers and drivers of speciation is discussed. Moreover, the importance of aggregation-sex pheromones as sexually selected signals is highlighted with evidence from the literature suggesting their potential role in species and mate recognition as well as mate assessment. The distinct evolutionary forces possibly involved are briefly reviewed and discussed in the context of this intriguing insect.

## Background

### A brief history of invertebrate sex pheromones

The first identification and published account of a sex pheromone (bombykol) was from the female silk moth, *Bombyx mori* in 1959 [1]. Karlson & Luscher [2] formally defined this new class of biologically active substances as pheromones (from the Greek “*pherein*”, to transfer and “*hormōn*”, to excite) and suggested a broad-based definition of pheromones as “*substances that are secreted by an animal to the outside and cause a specific reaction in a receiving individual of the same species, e.g., a release of certain behavior or a determination of physiologic development*.” [3]. Today there are more than 1,600 molecules described as sex pheromones spanning the majority of animal orders [4]; however the insects are by far the most prevalent taxon in the chemical world and exploit olfaction as their primary communication channel [5]. The definition of a sex pheromone remains somewhat contentious. Broadly defined, it is *any chemical that is emitted by one sex that elicits a response in the opposite sex*; however, this definition maybe too restrictive. Johansson & Jones (2007) expanded the original definition, defining a sex pheromone as *“any substance that is released by an individual, either directly from a specialized structure or that arises through changes in body chemistry, and that promotes subsequent variation in the sexual behaviour of individuals within the same species to the benefit of the releasing individual.”* [6]. It is this broader definition of sex pheromones that we adopt here, however we note that in cases where a sex pheromone has a dual function, for example, to promote mating aggregations it may be necessary to further sub-classify the chemical; in this instance as an *aggregation-sex* pheromone, *sensu* [7].

Over the past 57 years, our functional and mechanistic understanding of sex pheromones and pheromone communication has been transformed [5, 6, 8, 9]. Advancements are, in part, due to the increased sensitivity of detection and technologies surrounding accurate measurement. To place it into context the successful isolation of bombykol required 500,000 female moths; while today such analyses can be achieved with just a few or even single moths [10]. Precise measurement of the quantity and quality of chemicals emitted by single individuals has revealed considerable individual variation in both the composition and release rates of pheromones [6].

The majority of pheromones, regardless of their function, are comprised of more than one active component and they are typically species- and sometimes population-specific. To operate effectively and convey specific information, a pheromone is required to provide a message that permits accurate discrimination. This is particularly pertinent if there are multiple sympatric species using pheromones as their primary mode of communication. The specificity of the message can be achieved in a multitude of ways including variation in overall composition, the presence and composition of stereoisomers [11, 12] or the ratio of specific components all of which may lead to qualitative and quantitative differences in the signal emitted [5, 13, 14]. Even subtle changes in the pheromone blend (the specific ratios of chemicals within a pheromone) [15] or partitioning of communication channels through temporal or seasonal differences in pheromone production and emission as well as shifts in circadian activity [16–18] may result in individuals being unable to detect one another [9, 18, 19] and thus lead to speciation. Other factors such as the interaction with host produced volatile chemicals and preferences for particular habitats could also contribute to serve as mechanisms that lead to the avoidance of cross attraction between closely related heterospecifics that exist in sympatry [18].

Theoretical and empirical analyses of the evolution of sex pheromones suggest that pheromone blends evolve in one of two distinct and context-dependent ways [5]. The first proposes a gradual process of incremental changes in the pheromone blend, such as the loss or gain of single components, or variation in their relative proportions over evolutionary time. This hypothesis predicts that chemical signals are: highly conserved, are maintained through stabilizing selection, and are largely resistant to change. This mode of evolution results in phylogenetic conservatism, with closely related species having similar, or even identical, pheromone compositions. The alternate view suggests that pheromones, far from being conserved and stable, evolve rapidly via large saltational shifts [5]. Such saltational shifts generate a phenotype that either differs greatly or is completely different from the antecedent and may result in sibling species having highly dissimilar pheromones reducing the likelihood of interspecific responses. However, even when the pheromones of related species exhibit considerable differences in chemical composition, many compounds of pheromone blend have structural similarities, indicating that they share biosynthetic pathways [20].

As a signal, pheromones may be complex and multi-faceted and they can have multiple meanings depending on their context. Accordingly, sex pheromones have evolved and are maintained for a range of different functions such as species recognition, mate recognition and mate assessment [6, 21]. In species recognition, pheromones are used to discriminate between hetero- and con-specifics (members of the same species), thus interspecific competition for communication channels and selection for pre-mating reproductive isolation are the primary driving evolutionary forces. Pheromones involved in mate recognition indicate sex and reproductive status, and can advertise female receptivity for example. The environment is the predominant factor affecting the evolution of this kind of signal. Finally, mate assessment pheromones advertise the identity of the sender and its potential quality as a mate and, assuming this signal is honest (see below), they are predicted to be highly variable between individuals [6]. Theoretical contributions on the evolution of pheromones and empirical studies of pheromone-mediated mate choice also support the notion that sex pheromones can act as indicators of mate quality and are indeed used in individual mate assessment [21]. To be adaptive, mate assessment pheromones are predicted to correlate either directly or indirectly with traits such as condition, fertility, female reproductive status, age, parasite load, nutritional status, maturity, immunocompetence [6, 21] or inbreeding status [22]. These are mutually non-exclusive levels of mate choice and may be viewed as a continuum ranging from sexual isolation between species to individual mate assessment.

Arguably, to best understand the importance or role of chemical divergence in the process of speciation it is necessary to study groups, such as species complexes where gene exchange in nature is still occurring [6]. Here, we review the studies that contribute to the history of aggregation-sex pheromones within the phlebotomine sand fly, *Lutzomyia longipalpis* (Lutz & Neiva) species complex. We discuss the evolutionary forces that may be acting to maintain the integrity of the pheromone and highlight where future studies and investigations are required to better facilitate our understanding of the underlying mechanisms maintaining the observed variation in their aggregation-sex pheromone.

### The sand fly *Lutzomyia longipalpis*

#### Epidemiological importance

Visceral leishmaniasis (VL) is a vector-borne parasitic disease of significant medical importance because it can be lethal, it is difficult to treat and there is currently no vaccine [23]. It is estimated that 200,000–400,000 new cases of VL occur worldwide annually and over 90 % of new cases occur in just six countries: Bangladesh, Brazil, Ethiopia, India, South Sudan and Sudan. The death rate due to this disease is estimated to be 20,000 to 40,000 per year [24]. The sand fly *L. longipalpis* (Diptera: Psychodidae) is the main vector of American visceral leishmaniasis (AVL). Females, but not males are haematophagous, requiring and feeding on vertebrates’ blood to complete their gonotrophic cycle leading to the transmission of *Leishmania (Leishmania) infantum* (Nicolle) the etiological agent of the disease AVL [25–27].

*Lutzomyia longipalpis* has a wide geographical distribution in the Americas, occurring from Mexico to Argentina, and is found in a range of different habitats and diverse ecological conditions [25, 28]. Over the past 30 years, a new scenario has emerged as *L. longipalpis* has extended its natural range and adapted to domiciliary habitats in urban areas throughout Brazil, resulting in an increase in the incidence of both canine and human visceral leishmaniasis [26, 29]. Given its epidemiological significance a thorough understanding of the ecology and life history of the *Lutzomyia* species complex is critical. Advancement in this area has been pioneered by studies on pheromonal communication; indeed, such studies provide pivotal evidence for the existence of the *L. longipalpis* species complex.

### The *Lutzomyia longipalpis* species complex

**Fig. 1:**
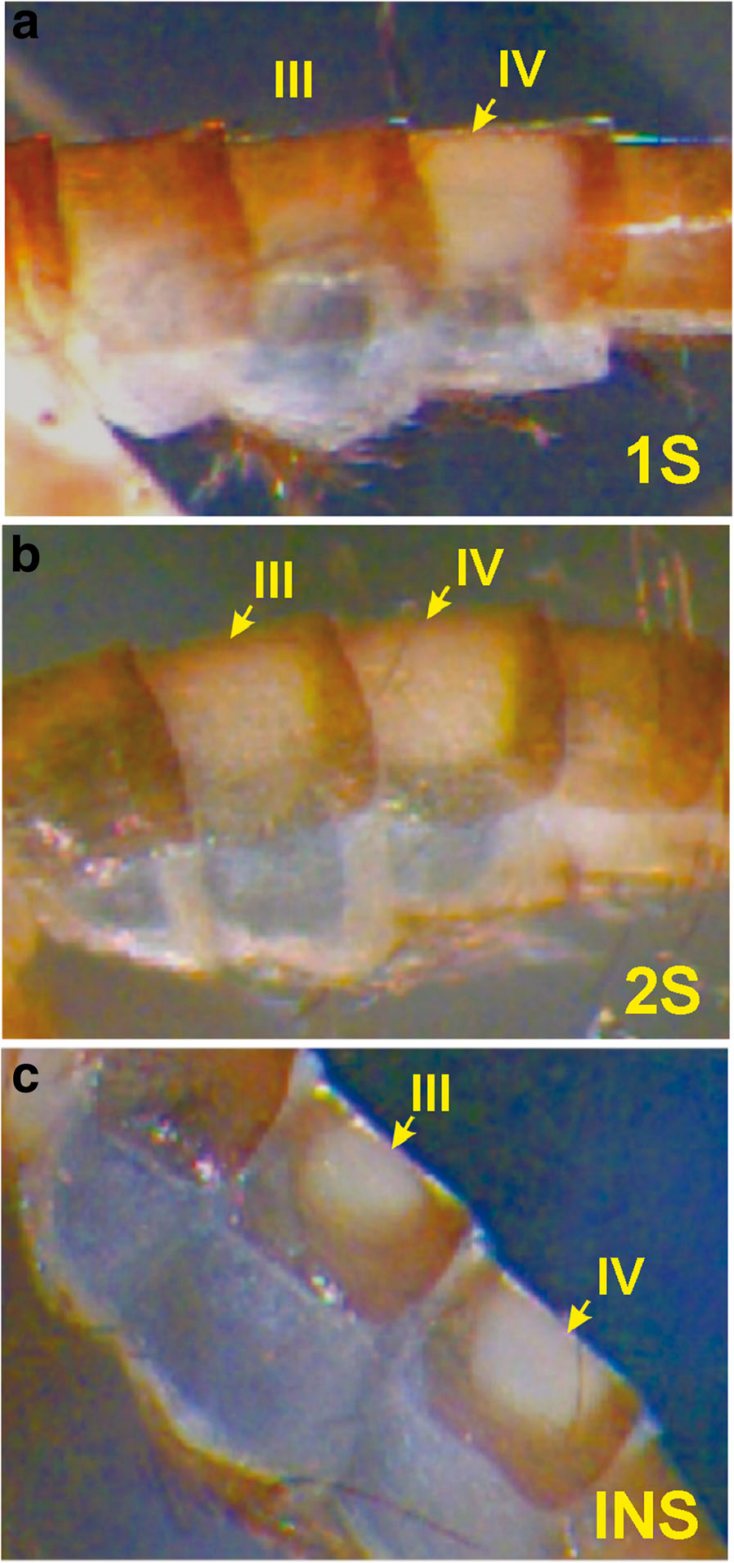
Morphological variation in the abdominal tergal pale spot patterns in males of *Lutzomyia. longipalpis*. **a** Single pale spot on the fourth abdominal tergite (phenotype named one spot phenotype, 1S). **b** Two pale spots on the third and fourth abdominal tergites (phenotype named two spot phenotypes, 2S). **c** Intermediate forms with one whole spot on the fourth abdominal tergite and a half spot on the third tergite (phenotype named intermediate form phenotype, INS). *Yellow arrows* indicate the pale spot

The first suggestion that *L. longipalpis* may be a sibling species complex came from the observed polymorphism in the number of abdominal spots on males [30]. All *L. longipalpis* males exhibit pale spots on their abdominal tergites; however, some populations have a single pair on the fourth tergite (one spot phenotype, named 1S), while others have two pairs on the third and fourth tergites (two spot phenotype, named 2S). In a few natural populations, there are also phenotypes in which the size of the tergal spot on the third segment shows considerable variation; the so-called intermediate forms (INS) (Fig. 1).

Scanning electron microscopy analysis of the tergites demonstrated the presence of numerous cuticular papules with central pores only on tergites with pale spots [31], suggesting that these might be sites of pheromone release. Further analysis revealed that the spot formation commences in the pupal phase and that, in the tergites where the aggregation-sex pheromone disseminating structures were located, the macrotrichia were absent perhaps facilitating pheromone dispersion [32]. Lane & Bernardes [33] used transmission electron microscopy of the tissue beneath the pale spots and showed a group of large columnar cells with an end apparatus connected to the exterior via a small cuticular duct, typical of gland cells. These were similar to the *Lutzomyia* male “odoriferous gland” described by Barth in 1961 [34], who first suggested that these glands were involved in stimulating the female prior to copulation. Unequivocal evidence for the role of these glandular areas in the production of sex pheromones in *L. longipalpis* was provided by Ward et al. (1989), who impregnated filter paper disks with whole tergal gland extracts and subsequently demonstrated that females were attracted over distances of up to 60 cm [35].

Crossing experiments between sympatric and allopatric Brazilian populations of *L. longipalpis* with dissimilar pale spots patterns suggested some degree of reproductive isolation. However, there are instances of populations with the same phenotype that exhibit reproductive isolation and populations with different phenotypes that do not [36–38]. The relationship between the number of pale spots and reproductive isolation was clarified when gland extracts were analyzed using coupled gas chromatography mass spectrometry [39, 40]. These analyses provided the first conclusive proof that male semiochemicals varied between different populations. Specifically, males were described as producing either a “farnesene/homofarnesene-like” molecule (C_16_H_26_) or “diterpenoid-like” molecule (C_20_H_32_). The reproductive compatibility of these populations correlated with the type of terpenoid compounds present was not associated with the spot phenotype [37–39], insects could share the same number of pale spots but the main component of the pheromone could vary. However, the reproductive incompatibility [37] between different populations was not fully explained by the presence of only 2 chemotypes.

**Fig. 2:**
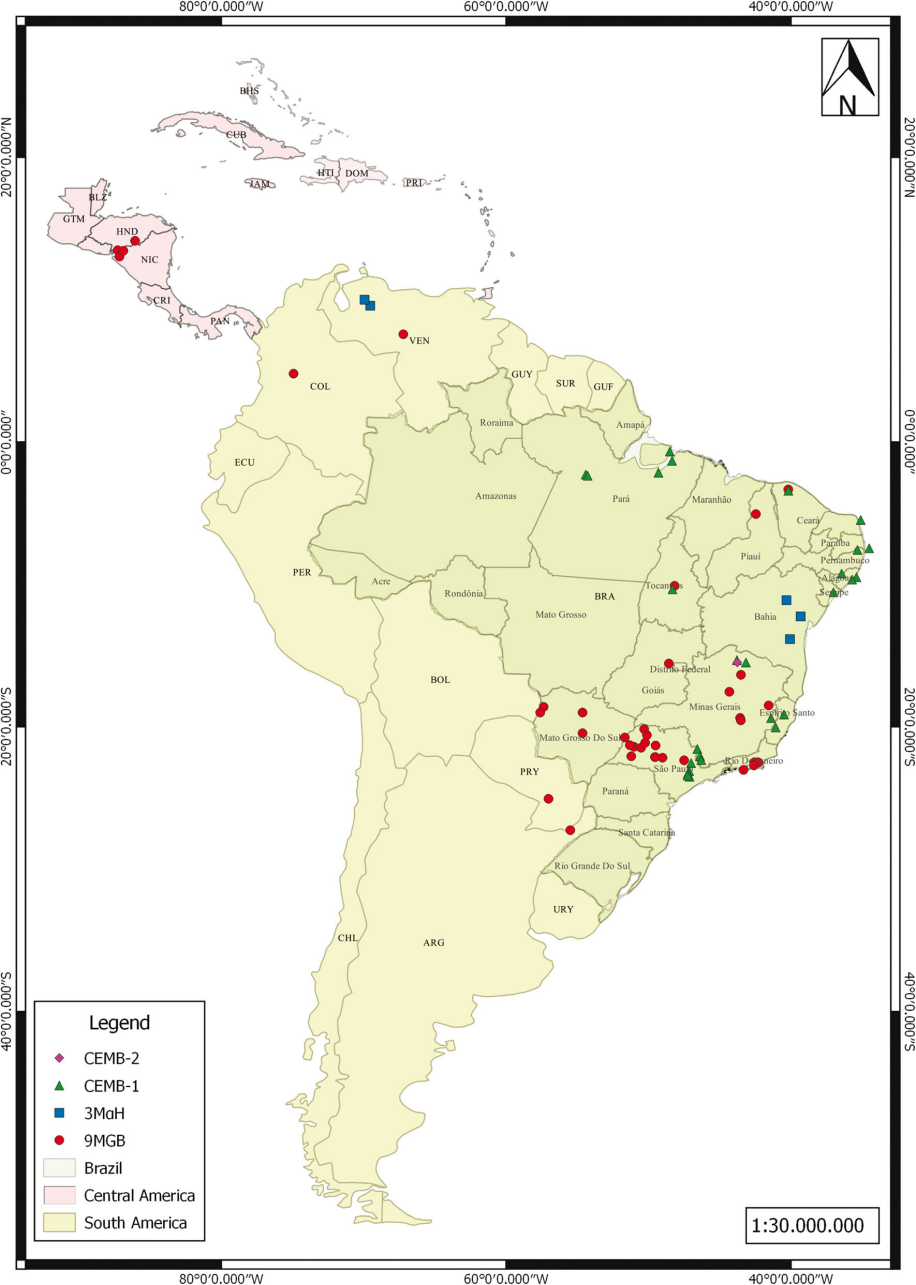
Geographical locations of the different *Lutzomyia longipalpis* aggregation-sex pheromone chemotypes in Americas. This map was created using Qgis Pisa version 2.10.1, coordinate system: SAD 69 and database: ZEE/AC, 2006

Laboratory assays showed that the main component of an extract of the tergal spots was responsible for most of female’s attraction and the minor components only marginally enhanced the attraction of the major component [41]. This initial behavioral analysis was carried out for the Jacobina (Bahia State, Brazil) population of *L. longipalpis* where the main component makes up 90 % of the compounds found in the tergal gland extract. Based on the analysis of the main terpene component of the tergal gland extract, four distinct chemotypes can now be recognized in the *L. longipalpis* complex: chemotype 1 or (*S*)-9-methylgermacrene-B (9MGB) found for example, in several Brazilian States such as Minas Gerais, Piauí, Rio de Janeiro and Sao Paulo, as well as in other countries such as Argentina, Colombia, Paraguay, Honduras and Venezuela, chemotype 2 or (*1S,3S,7R*)-3-methyl-α-himachalene (3MαH) in Jacobina (Bahia State) also found in *L. pseudolongipalpis* (Venezuela), chemotype 3 or cembrene-1 (CEMB-1) in Sobral 2S (Ceará State), Santarém (Pará State), Estrela de Alagoas 1S and 2S (Alagoas State), Costa del Sol (Alagoas State), Pancas (Espírito Santo State) and Jaíba 2S (Minas Gerais State), and chemotype 4 or cembrene-2 (CEMB-2) in Jaíba 1S (Minas Gerais State) [42–49]. Potentially, a fifth chemotype has been identified based on variation in the amount of specific terpenes present, as well as morphological differences: chemotype 5 (or 9MGB+) found in Sobral 1S, Sobral INS (Ceará State) and Montes Claros (Minas Gerais State) [46]. Currently, we consider chemotype 5 to be analogous to chemotype 1, based only on the main component of the pheromone. However, sex pheromone specificity may depend on a range of factors such as differences in chain length, position of double bonds, stereo configuration or variation in the ratios of different components and thus understanding the significance of quantitative and qualitative variation in total terpenes is essential. Further work is required to confirm the epidemiological and evolutionary appropriateness of collapsing chemotype 5 into chemotype 1; however such studies are logistically challenging [46]. A map of the distribution of currently identified chemotypes within the *L. longipalpis* complex in the Americas (as well as some related species) are presented in Fig. 2 and Table 1. Notable is the fact that chemotype 1 (9MGB) was also identified in *Lutzomyia cruzi* (Mangabeira, 1938) [50], a sibling species that may turn out to be a part of the *L. longipalpis* complex [51]. The possibility that they are isomers has not been investigated by more refined analyses (Hamilton JG, Brazil R., Personal communication, 2015).

**Table 1:** Distribution of the different chemotypes described in America

Chemotype	Pheromone	Species	Location	State	Country	Reference
1	9MGB	*L. cruzi*	El Carmen		BO	Hamilton & Brazil (Personal communication, 2015)
	9MGB	*L. cruzi*	Corumbá	Mato Grosso do Sul	BR	[47, 50]
	9MGB	*L. cruzi*	Ladário	Mato Grosso do Sul	BR	[47]
	9MGB	*L. longipalpis*	Guayabita	Aragua	VE	[47]
	9MGB	*L. longipalpis*	Vila Elisa	Asunción	PA	[114]
	9MGB	*L. longipalpis*	Sobral	Ceará	BR	[47]
	9MGB	*L. longipalpis*	Orocuina	Choluteca	HO	[43]
	9MGB	*L. longipalpis*	Pavana	Choluteca	HO	[43]
	9MGB	*L. longipalpis*	San Juan Bautista	Choluteca	HO	[43]
	9MGB	*L. longipalpis*	Tololar	Choluteca	HO	[43]
	9MGB	*L. longipalpis*	Pirenópolis	Goias	BR	Hamilton & Brazil (Personal communication, 2015)
	9MGB	*L. longipalpis*	El Layero	Guárico	VE	[47]
	9MGB	*L. longipalpis*	São Luís	Maranhão	BR	Hamilton & Brazil (Personal communication, 2015)
	9MGB	*L. longipalpis*	3 lagoas	Mato Grosso do Sul	BR	Hamilton & Brazil (Personal communication, 2015)
	9MGB	*L. longipalpis*	Campo Grande	Mato Grosso do Sul	BR	[29]
	9MGB	*L. longipalpis*	Belo Horizonte	Minas Gerais	BR	[51]
	9MGB	*L. longipalpis*	Governador Valadares	Minas Gerais	BR	Hamilton & Brazil (Personal communication, 2015)
	9MGB	*L. longipalpis*	Lapinha Cave	Minas Gerais	BR	[42, 46, 47]
	9MGB	*L. longipalpis*	Montes Claros	Minas Gerais	BR	[46]
	9MGB	*L. longipalpis*	Lassance	Minas Gerais	BR	[51]
	9MGB	*L. longipalpis*	Posadas	Misiones	AR	[115]
	9MGB	*L. longipalpis*	Teresina	Piauí	BR	[48]
	9MGB	*L. longipalpis*	Rio Bonito	Rio de Janeiro	BR	[29]
	9MGB	*L. longipalpis*	Niterói	Rio de Janeiro		Hamilton & Brazil (Personal communication, 2015)
	9MGB	*L. longipalpis*	Barra de Guaratiba	Rio de Janeiro	BR	[48]
	9MGB	*L. longipalpis*	Saquarema	Rio de Janeiro	BR	Hamilton & Brazil (Personal communication, 2015)
	9MGB	*L. longipalpis*	Adamantina	São Paulo	BR	[29]
	9MGB	*L. longipalpis*	Araçatuba	São Paulo	BR	[29]
	9MGB	*L. longipalpis*	Bauru	São Paulo	BR	[29]
	9MGB	*L. longipalpis*	Dracena	São Paulo	BR	[29]
	9MGB	*L. longipalpis*	Jales	São Paulo	BR	[29]
	9MGB	*L. longipalpis*	Lourdes	São Paulo	BR	[29]
	9MGB	*L. longipalpis*	Marília	São Paulo	BR	[29]
	9MGB	*L. longipalpis*	Oswaldo Cruz	São Paulo	BR	[29]
	9MGB	*L. longipalpis*	Presidente Prudente	São Paulo	BR	[29]
	9MGB	*L. longipalpis*	Promissão	São Paulo	BR	[29]
	9MGB	*L. longipalpis*	Salmourão	São Paulo	BR	[29]
	9MGB	*L. longipalpis*	Porto Nacional	Tocantins	BR	[126]
	9MGB	*L. longipalpis*	L’Aguila	Tolima	COL	[127]
	9MGB + CEMB-1	*L. longipalpis*	São Pedro	São Paulo	BR	[29]
2	3MαH	*L. longipalpis*	Cavunge	Bahia	BR	Hamilton & Brazil (Personal communication, 2015)
	3MαH	*L. longipalpis*	Jacobina	Bahia	BR	[41, 44]
	3MαH	*L. longipalpis*	Jequié	Bahia	BR	Hamilton & Brazil (Personal communication, 2015)
	3MαH	*L. pseudolongipalpis*	La Rinconada	Curarigua Lara	VE	[47]
	3MαH	*L. pseudolongipalpis*	El Paso	Lara State	VE	[116]
3	CEMB-1	*L. longipalpis*	Afonso Cláudio	Espírito Santo	BR	Hamilton & Brazil (Personal communication, 2015)
	CEMB-1	*L. longipalpis*	Águas da Prata	São Paulo	BR	[29]
	CEMB-1	*L. longipalpis*	Barcarena	Pará	BR	Hamilton & Brazil (Personal communication, 2015)
	CEMB-1	*L. longipalpis*	Camará	Pará	BR	Hamilton & Brazil (Personal communication, 2015)
	CEMB-1	*L. longipalpis*	Campinas	São Paulo	BR	[29]
	CEMB-1	*L. longipalpis*	Espírito Santo do Pinhal	São Paulo	BR	[29]
	CEMB-1	*L. longipalpis*	Estrela de Alagoas	Alagoas	BR	[46]
	CEMB-1	*L. longipalpis*	Indaiatuba	São Paulo	BR	[29]
	CEMB-1	*L. longipalpis*	Ipanema	Minas Gerais	BR	Hamilton & Brazil (Personal communication, 2015)
	CEMB-1	*L. longipalpis*	Jaíba	Minas Gerais	BR	[45]
	CEMB-1	*L. longipalpis*	Maceió	Alagoas	BR	Hamilton & Brazil (Personal communication, 2015)
	CEMB-1	*L. longipalpis*	Marajó	Pará	BR	[47]
	CEMB-1	*L. longipalpis*	Natal	Rio Grande do Norte	BR	[47]
	CEMB-1	*L. longipalpis*	Pancas	Espírito Santo	BR	[48]
	CEMB-1	*L. longipalpis*	Passira	Pernambuco	BR	Hamilton & Brazil (Personal communication, 2015)
	CEMB-1	*L. longipalpis*	Porto Nacional	Tocantins	BR	[126]
	CEMB-1	*L. longipalpis*	Salto	São Paulo	BR	[29]
	CEMB-1	*L. longipalpis*	Santarém	Pará	BR	[46]
	CEMB-1	*L. longipalpis*	Sobral	Ceará		[46]
	CEMB-1	*L. longipalpis*	Socorro	São Paulo	BR	[29]
	CEMB-1	*L. longipalpis*	Sol da Costa	Alagoas	BR	[46]
	CEMB-1	*L. longipalpis*	Sorocaba	São Paulo	BR	[29]
	CEMB-1	*L. longipalpis*	Votorantim	São Paulo	BR	[29]
	CEMB-1	*L. longipalpis*	Aracajú	Sergipe	BR	Hamilton & Brazil (Personal communication, 2015)
	CEMB-1	*L. longipalpis*	Cametá	Pará	BR	Hamilton & Brazil (Personal communication, 2015)
	CEMB-1	*L. longipalpis*	Itamaracá	PE	BR	Hamilton & Brazil (Personal communication, 2015)
	CEMB-1	*L. longipalpis*	Nova Porteirinha	Minas Gerais	BR	Hamilton & Brazil (Personal communication, 2015)
4	CEMB-2	*L. longipalpis*	Jaíba	Minas Gerais	BR	[45]

The best evidence for the existence of a *L. longipalpis* complex comes from observations of species coexisting in sympatry. Hamilton’s et al. [46] comprehensive analysis of individual males of three different spot phenotypes (1S, 2S and INS) from Sobral suggests that there are probably two sympatric chemotypes (3 and 5) corroborating previous findings [36, 52]. Mating-crosses between individuals from Lapinha Cave (9MGB, chemotype1) and Jacobina (3MαH, chemotype 2) yielded male offspring with both 9MGB and 3MαH (Hamilton & Rebollar-Tellez, unpublished). The absence of chemical hybrids of the 9MGB and CEMB-1 chemotypes in the individually analysed male samples from Sobral suggests that those two chemotypes are reproductively isolated. Crossing experiments carried out between the two Sobral chemotypes indicate both copulatory and pre-mating isolation [37]. In simple laboratory choice experiments individual Jacobina population females were attracted only to the conspecific pheromone and Sobral 2S females given a choice showed a preference for conspecific male pheromone, however they also responded to Jacobina male pheromone [53]. In contrast, field studies that used traps baited with pheromone from two different pheromone chemotypes (either 9MGB from Araçatuba, Sao Paulo State or CEMB-1 from Marajo, Para State) found that both male and female sand flies were significantly more attracted to their conspecific pheromone chemotype compared to the heterospecific chemotype. Combined these data provide strong support for the argument that the aggregation-sex pheromones act as an important pre-mating isolation barrier and may have diversified through selection operating within the *L. longipalpis* complex speciation [54].

Two general conclusions can be drawn from these studies: first, the spot phenotype cannot be used as a reliable marker to identify different species (although there are coincidental exceptions, e.g. in Sobral); secondly, the terpene profile of allopatric populations may be qualitatively the same as sympatric populations (e.g. CEMB-1 producing populations from Estrela de Alagoas, Sol da Costa and Santarém *vs* Sobral 2S) [46]. However, currently unrecognized taxonomic substructures could exist within these chemotypes: for example, there are significant differences in the quantities of terpene (Chemotype 1) produced by Lapinha Cave and Sobral 1S/INS (Chemotype 5) populations and also between the Chemotype 3 Sobral 2S and Santarém/Sol da Costa/Estrela de Alagoas populations.

Many insect semiochemicals are chiral compounds where the biological activity of each enantiomer differs, where the “unnatural” enantiomer (that not produced by the insect) may be equally active, less active (but enhancing the activity of the natural isomer) or even inhibiting the activity of the active isomer [11]. Our understanding of the stereospecificity of either CEMB-1 or CEMB-2 molecules is incomplete. All *L. longipalpis* collected in Estrela de Alagoas produced the same cembrenes regardless of spot type [46], acoustic differences and genetic background [48]. This may arise because the pheromones differ in the kind of CEMB-1 isomer expressed within each population. More detailed analysis is required to better reveal the true diversity of chemotypes within *L. longipalpis* species complex and perhaps more importantly their evolutionary significance.

The taxonomic status of *L. longipalpis* has been the focus of a number of studies using populations distributed over a broad geographical range spanning countries in Central and South America [55, 56]. Several independent studies using different approaches support the species complex hypothesis in Latin America. In contrast, evidence from Brazilian populations is contradictory: depending on the molecular marker used, some studies estimated genetic distances compatible with a single species and others support the species complex hypothesis [57, 58]. Differential rates of evolution among genes, maintenance of ancestral polymorphisms and introgression are factors that describe discordant evolutionary history, especially for very closely related species such as *L. longipalpis* complex [59]. Microsatellites and nuclear genes associated with sexual behavior in *Drosophila* such as *cacophony*, *paralytic* and *period* (*per*) genes are among the markers that have proven useful for identifying clusters of putative species [47, 48, 51, 52, 57, 58, 60–63].

Furthermore, in addition to variation in sex-pheromone type [38, 43, 46] the existence of a species complex has been proposed based on results of analyses of the male courtship song [64, 65]. The courtship song is produced by *L. longipalpis* during pre-mating courtship and copulation [37, 48] and may play a role in species recognition [66, 67]. Copulation song analyses of *L. longipalpis* populations from around Brazil revealed at least six basic song types. Laboratory crosses between some of these different song types populations resulted in insemination failure [37, 38]. An integrative study including data on copulation song, sex pheromone and molecular variation of *per* gene yielded two main groups: a homogeneous group producing a burst-type and CEMB-1 and a heterogeneous group producing five different pulse-song patterns (designed as P1 to P5) and four different pheromones (9MGB, 3MαH, CEMB-1 and CEMB-2) [48]. A multilocus approach estimated that the two lineages split about 0.5 million years ago [59]. However, the evidence of introgression, suggest a posterior secondary contact in localities such as Sobral and probably indicate that the splitting time was not long enough ago to ensure the appearance of full reproductive isolation mechanisms. More recently, a study using a more comprehensive geographical sampling regime corroborates the existence of at least six species in Brazil based on copulation song parameters [68].

### The significance of pheromone for *Lutzomyia longipalpis*

While the evolution of a chemical cue is a chance event, its maintenance in the population can only be sustained if its presence yields adaptive benefits, or at least if its costs are neutral. In *L. longipalpis* the male produced pheromone is (either directly or indirectly) implicated in species- and mate-recognition as well as in mate assessment.

#### Not just a sex pheromone

Male sand flies of the *L. longipalpis* species complex form crepuscular and nocturnal aggregations (or leks), which persist for many hours, on or near vertebrate hosts [69–71]. Males are attracted to host odours (kairomones) [72] even though, in contrast to females, they are not haematophagous. Field-based studies using traps demonstrated that males arrive earlier at host-sites and that their arrival is related to both host- and male-abundance [70]. Females visit leks to obtain a blood meal and to mate and are attracted to both the host kairomones and the male-produced sex pheromone [73, 74]. There is temporal separation in the arrival of males and females: females arrive later and stay for shorter periods and both female immigration and abundance are related to the distribution of males rather than the distribution of hosts [75]. In contrast, male emigration rates from the lek are inversely related to host and fly abundance suggesting that they are using semiochemicals to maintain aggregations [70]. Males also appear to retain their position at the lek in a given night and return to the same site over multiple nights [70].

Dougherty et al. [76] made the first electrophysiological recordings from ascoid sensillae on the female antennae and reported neurons sensitive to the sex pheromone. Ascoid sensillae are paired structures found on the 3rd-15th antennal segments, in both sexes [77]. In a laboratory study, that coupled capillary gas chromatography with electrophysiological recordings from ascoid sensillum receptor cells it was demonstrated that receptor cells from both male and female ascoid sensillae responded only to the major component (3MαH) of the sex gland extract of *L. longipalpis* from Jacobina Brazil [78]. Behavioral experiments also confirmed that both males and females from the Jacobina population flew upwind in a wind tunnel towards a filter paper disk treated with tergal gland extract, pure (*1S,3S,7R*)-3-methyl-α-himachalene or the synthetic mixture of eight isomers thus supporting the proposal that 3MαH derived from *L. longipalpis* Jacobina males has a dual function in promoting male aggregations as well as serving as a sex pheromone for females. Subsequent field studies with a different chemotype corroborated this proposal as synthetic pheromone traps containing a racemic mixture of 9MGB attracted flies of both sexes [79] even when used in conjunction with insecticide sprayed animal houses [80].

**Fig. 3:**
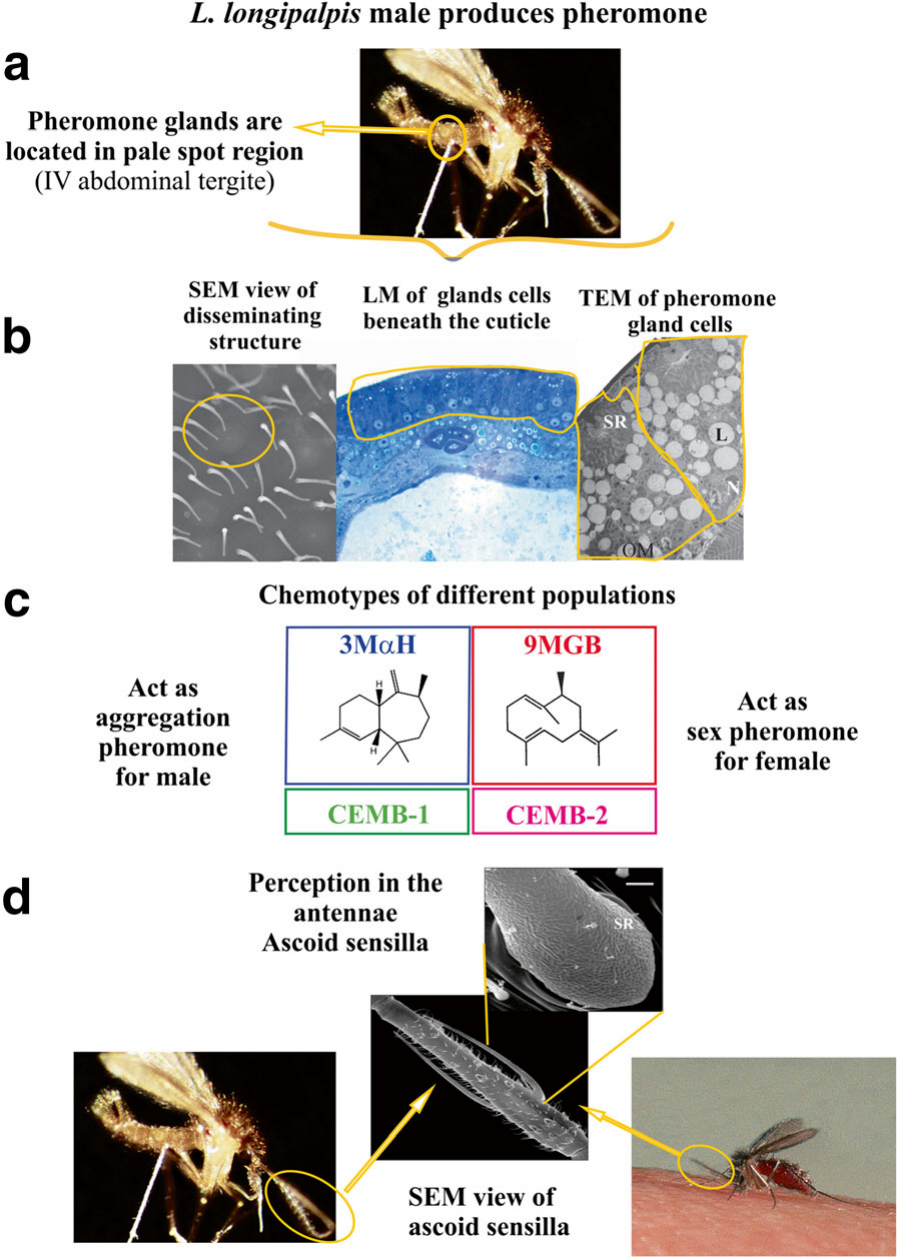
*Lutzomyia longipalpis* aggregation-sex pheromone communication system. **a** The aggregation-sex pheromones is produced by males in glands located in the pale spots in the third and/or fourth abdominal tergites. **b** Scanning Electron microscopy (SEM) of the cuticular papules with a central pore disseminating structures located in pale spots. The secretion is produced by pheromone gland cells grouped beneath the cuticle showed in light microscopy (LM). Details of two large columnar secretory cells can be observed in this transmission electron microscopy (TEM). Each gland cell is connected to the exterior via a small cuticular duct and present two distinct parts: a basal region with vacuoles containing lipids (L) and an apical region with an end apparatus and a small reservoir (SR). **c** The main component of the pheromone differs among populations and represent different chemotypes. **d** The pheromone is perceived by the paired ascoid sensilla present in antennae of both male and female showed in SEM. The pheromone functions for male as an aggregation pheromone and as sex pheromone by female

When combined, the empirical data suggests that the glandular sex pheromone functions to promote both aggregation of males and attraction of females. This represents both the first and possibly second level of mate choice: namely, species recognition and sex identification. The final level of choice is individual identification and the degree to which pheromones contribute to female mating preference is less well understood. It is conceivable that a male’s mating success is directly related to pheromone gland content [81], however it may be related to other traits, such as courtship behavior, copulatory courtship song [68] or other pheromones such as cuticular hydrocarbons (CHCs) [82]. In practice it is likely that multiple factors contribute to male mating success, however if pheromones are utilized in mate choice then theoretically it is predicted they should be honest signals and thus most likely to be costly to produce. An overview of the *L. longipalpis* sex-aggregation pheromone communication system from a pheromone biosynthesis perspective and showing the different chemotypes produced and the apparatus involved in perception of pheromone can be seen in Fig. 3.

#### Individual variation in pheromone production

Early behavioral studies revealed variation in male mating success related to age and although not explicitly demonstrated, this may relate to variation in pheromone production [81]. Recent chemical analyses of age-related pheromone content in males confirm a gradual increase in pheromone production in synchrony with the pheromone gland cells maturation [83]. This study did not examine the pattern in even older males, thus whether a decline in pheromone content commensurate with senescence is unknown [81]. Nonetheless, age-related signal degradation is one mechanism that honesty can be maintained within the system and this may be adaptive in *L. longipalpis* because on average, older males sire fewer viable offspring [81]. It is interesting to note that in *L. longipalpis* males, the genitalia undergo a 180° rotation after emergence, taking 24 h for the insects to become sexually mature [28] and pheromone biosynthesis commences around 12 h after emergence, increasing until three days of age and then stabilizing thereafter [83]. Noteworthy also then, is the fact that *L. longipalpis,* males commence the adult stage of their lives with no ability to attract females, at least from a distance and, if the pheromone signal deteriorates with old age, they are similarly less capable of attracting females as they senesce. Indeed, the nature of signal production in *L. longipalpis* may be one of the key reasons why a lek-mating aggregation strategy is adaptive for both sexes. Younger males (who are incapable of high pheromone output) and older males (if their signal does indeed deteriorate with age) might benefit from parasitizing the communication output of average-age individuals and females are provided with a choice of males [84]. Whether female mating preferences are related directly to the sex pheromone or also include CHC profiles (as has been demonstrated for other insects [85–87]) is unknown but their presence has been proposed in *L. longipalpis* [82, 88] and has been demonstrated for related sand fly species [89–93]. However, whether or not some of those CHC have pheromonal properties is not known. Selecting for specific lines based on chemical traits are untested in *L. longipalpis* but this might prove a way of determining the significance of the direction and force of selection on pheromones and chemoreception.

#### Cost of production - sexual selection

Understanding the costs of pheromone production remain largely elusive across invertebrates and whether signals that are involved in reproduction, and have presumably evolved through sexual selection, are required to be costly to be honest remains a subject of considerable debate (for a recent comprehensive review see, Steiger & Stökl [21]). Some chemical signals might be expressed in relation to body condition or vary with age; others may more reliably reflect an individual’s genetic quality. The costs of production are rarely demonstrated in insects (but see Johansson et al. [94]), however these can be inferred indirectly in species where signal production commences after adult eclosion (as is the case for *L. longipalpis* [83]). Recent transcriptomics and proteomics studies have revealed much about the biosynthetic pathway for glandular secretions in *Lutzomyia* including the specific enzyme pathways involved in sesquiterpenoid biosynthesis [95, 96]. It was observed in *L. longipalpis* that if the diet is changed, e.g. glucose to fructose, there is a change in the amount of pheromone produced (Hamilton, unpublished). These results might suggest that pheromone production will be optimal if diet is optimal.

### Distinct evolutionary forces acting on *L. longipalpis* aggregation-sex pheromones

A key question that remains is why the pheromone composition varies across populations? The most parsimonious explanation is that it provides an efficient means by which closely related sympatric subspecies can limit hybridization [52, 59]. Only a small number of hybrids exist in sympatric populations in nature [29, 46]. Field [54] and laboratory [36–38] data indicate that Brazilian populations of *L. longipalpis* respond to the male pheromone in a sibling species-specific manner. Therefore, the male sex pheromones may act as a pre-mating isolation barrier, reducing non-productive mating encounters and therefore may be important influences on speciation in the *L. longipalpis* complex. However, the evidence on whether the chemical cues observed are sufficiently isolating is not strong and the evidence that currently exists suggests that short-range behavioral traits such as courtship [82] or songs [59, 64, 68] play an important additional species isolating role. From an evolutionary perspective it is likely that chemical shifts may drive significant reproductive diversification and ultimately speciation [5, 9].

Current evidence regarding temporal variation in release is also limited. Ultrastructural analysis of male unicellular sex pheromone glands beneath the cuticle shows a small structure, the end apparatus surrounded by secretory cells that become highly vacuolated as the male sand fly matures. Therefore, the potential to accumulate pheromones in cuticular glands is small but the rather larger amounts of lipid containing vaculoles suggests that although pheromone production and release may be not be instantaneous but more gradual [32]. Small, but potentially significant variation in locomotor activity is observed across sympatric populations of *L. longipalpis* [97], however further studies exploring simultaneously circadian rhythms and pheromone release are necessary to exclude the hypothesis of some kind of temporal partitioning of communication channels such as that observed in moths [16, 98].

The direction of selection for pheromone output has not been tested explicitly in *L. longipalpis* but several studies suggest that males benefit from attending aggregations (because larger leks attract more females) and that females also gain indirect benefits through a greater number of mate choice options. The evolution of a male pheromone that attracts other males is only sustainable if this also leads to an increase in male reproductive success, unless the production of that pheromone was cost neutral. The fact that males take multiple days to achieve maximal pheromone output suggests that there are likely costs and thus neutrality cannot be invoked. Thus, we must assume that there are adaptive benefits to its production and they must include fitness benefits such as increased mating success and hence offspring production. Although attraction of other males does not seem to be beneficial for the emitter, it may evolve if the cost of “not calling” is larger than the cost of “having to share” the responding female [99]. Moreover, aggregations can facilitate mate-finding, resulting in energetic savings on search costs. However, male signaling in *L. longipalpis* may confer advantages to females other than just for mate finding, particularly because lek formation often occurs on or near hosts [69, 100] and thus females gain the necessary resources for offspring production. Laboratory experiments also suggest a synergistic effect (in terms of female attraction) when the sex-pheromone and the host odor are combined [73, 74, 101]. This is in accordance with Landolt’s hypothesis that mate-finding systems based on male-produced sex attractants are usually associated with feeding and oviposition sites and thus serve to compensate for the risks and increased energy expenditure associated with mate searching [102]. Accordingly, many species demonstrate an increased response to specific aggregation pheromones when they are accompanied by co-attractants [99]. The benefits that an individual male accrues from lekking are likely determined by his individual quality, whereby high-quality males in large leks gain access to more females [75]. In *L. longipalpis* females do not mate readily a second time (at least during a single gonotrophic cycle) [103]. Behavioral observations revealed a reduction in mating frequency of copulated females [103, 104] and transcriptome analyses from male sand fly reproductive organs that identify ESTs encoding orthologs of Seminal Fluid Proteins (SFPs) [105], indicate the presence of putative *L. longipalpis* SFPs which may reduce sexual mating frequency of copulated females (like in *Drosophila melanogaster*) [106, 107]. The presence of a mating plug has been observed in the spermatheca of other sand flies such as *P. perniciosus*, *P. papatasi* [108] and *P. duboscqui* [109]. While not explicitly tested it probably acts to reduce, if not prevent, multiple mating in females and is likely to be driven by male-imposed means through sexual conflict.

### Dispersion of populations with different pheromones

A gap in the current literature is that little is known regarding the evolutionary pathway of pheromone synthesis across the *L. longipalpis* species complex*.* In moths, changes in enzymes involved in the synthesis pathway of pheromones do produce different pheromones in closely related species [20, 110, 111]. In *L. longipalpis* it is conceivable that an analogous process is occurring. González-Caballero [95] and colleagues used transcriptome and proteome data to explore the enzymatic cascade from the sex pheromone gland. Molecules which are associated with enzymes of the mevalonate pathway were found in the pheromone gland. However, the later steps of production of isoprenoid compounds led to different end products from those found in other insects. Although there is little information available on the biosynthesis of cyclic sesquiterpene or methylsesquiterpene compounds in insects, mutations or other genetic events, at least in one enzyme might result in different pheromones. Recently the characterization of genes that cause qualitative changes in insect pheromones was described in detail for the Hymenoptera [112]. Elegant experiments provide support for the idea that new pheromone compounds can emerge from modifications in existing signaling molecules. Furthermore, and perhaps most interesting is that sex pheromone receptors do not appear to immediately respond to novel pheromone compounds within the existing pheromone blend. This provides a mechanism by which new pheromone components may initially escape from selection exerted by the receivers and, concomitantly, receptors would have a time to associate the new compound with conspecific mates and to be recognized it as part of the species-specific chemical signal [112].

Associations between Brazilian populations and Central and South America populations could indicate that the diterpene form has evolved from the widespread 9MGB type. To date, the diterpene producing-form has not been found outside of Brazil and all sex pheromones typed in Venezuela, Honduras, Guatemala, Colombia, Bolivia, Paraguay and Argentina, as well as several Brazilian populations (e.g. Lapinha, Teresina, Barra de Guaratiba) [38, 47, 113–115] were the 9MGB type. An exception in America is the *L. pseudolongipalpis* (also belonging to the *L. longipalpis* complex) that produces 3MαH, the same main component as the Jacobina *L. longipalpis* population from Brazil [116]. Clearly, the type of sex pheromone released by the males overrides geographical distance effects upon the phylogeographic structure of *L. longipalpis*. Based on ecological distribution alone, one would predict that 9MGB is the ancestral chemotype in *L. longipalpis* across the savannahs of South America, followed by subsequent speciation to either 3MαH/αH or CEMB [47].

*Lutzomyia longipalpis* has an extensive and patchy distribution from southern Mexico to Argentina, occurring in diverse ecological conditions such as dry habitats, humid forests [117], but also in urban areas [118]. It remains a challenge to understand the evolutionary history of this group and the mechanism underlying its success in dispersion and adaptation, despite its limited flight range [100, 119, 120]. How geographical barriers [121] and, more recently, anthropogenic environmental changes and activities have contributed to the evolution of sibling species remains a future challenge [26]. Molecular analyses and geological history [59, 121] will aid our understanding of populations distributions but these need to be coupled with studies of variation in traits involved in mating such as copulation songs [64, 65, 68] and chemical communication [29, 46, 47]. These latter factors have undoubtedly played (and continue to play) a significant role in maintaining reproductive isolation among the different sibling species [48, 55, 60].

Finally, it is unclear whether there are any general epidemiological implications associated with the observed variation in chemotype such as that hypothesized by Casanova [29]. An obvious disadvantage to rapid adaptation of the components of the sex pheromone is that this makes it far trickier to detect and/or control using synthetic chemical baits, unless the different chemotypes are key signature pheromones that operate within the species complex. Thus, a thorough understanding of the selective pressures and underlying mechanisms that generates shifts in the composition of the sex pheromone is clearly of paramount importance from both an evolutionary but an epidemiological perspective.

### Presence of papules in other sand fly species

**Fig. 4:**
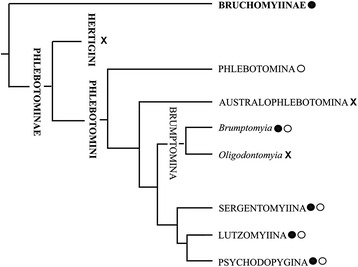
Phylogenetic tree of the family Psychodidae including the different subtribes of the subfamily Phlebotominae indicating the presence (•) or the absence (**o**) of papule in the abdominal tergites of different groups as well as the lack of studies (x). Modified from Galati et al. [123]

The subfamily Phlebotominae is very diverse and widely spread in the world, found in different ecological environments and continents, with more than 1,000 species known. The phylogenetic classification used by our group recognize different subdivisions within this subfamily which includes the presence or absence of papules in the abdominal tergites in the identification key [122, 123]. It is interesting to note that the papules were described by microscopy studies in different species of the family Psychodidae [32–34, 124, 125]. However, corresponding behavioral or chemical studies are largely absent. Given this, we are unable to conclude that papules always act as a pheromone disseminating structure, although a minority of studies indicates some relationship between the papule and the presence of pheromone. If we analyze among different groups of sand flies the presence of papules and put it in a phylogenetic tree, parsimony suggests that their presence is a primitive character and these papules were thus lost in some groups. Even in a more primitive Psychodidae group such as the Bruchomyinae the papules are present, indicating that their presence in more recent groups such as *Lutzomyia* is not exclusive or a novelty. Similarly, in the same subtribes of the subfamily Phlebotominae, we can find species with and without papules (Fig. 4), for example in the subtribes Brumptomyiina, Sergentomyiina, Lutzomyiina and Psychodopygina. It is unknown whether the tribe Hertigiini, the subtribe Australophlebotomina or the genus *Oligodontomyia* has papules; however limited evidence suggests that at least some species in the subtribe Phlebotomina do not have papules, but we cannot be sure if papules are absent in all species of this group or whether their absence reflects a lack of sampling effort. Due to the fragmented disposition of the papules across the different phylogenetic groups, it is challenging to understand why the presence of pheromones is not consistent particularly as they are so advantageous. Further studies are necessary to better understand the evolution and the loss of pheromone cues in different species of seemingly closely related Phlebotominae.

## Conclusions

Species complexes are important models for understanding the role of chemical divergence in speciation. Here, we used an evolutionary framework to review the history of aggregation-sex pheromones within the *L. longipalpis* species complex. In this group, aggregation-sex pheromones are produced by males during territorial courtship displays. The male produced pheromone (in synergy with host odors) attracts haematophagous females to a common site of copulation and feeding. Observations of variation in the sex-pheromone coupled with knowledge of geographical distribution of sandflies indicates four distinct chemotypes; (*S*)-9-methylgermacrene-B, the most widespread in South and Central America, followed by cembrene-1, cembrene-2 and (*1S,3S,7R*)-3-methyl-alpha-himachalene. It is possible that more taxonomic substructures exist within the current chemotypes: potentially, a fifth chemotype has been identified based on variation in the amount of specific terpenes present and our understanding of the stereospecificity of cembrene molecules remains incomplete. Although those chemotypes exist in sympatry, the chemical hybrids which can be generated in laboratory experiments, are rare in nature. Field and laboratory studies show that there is no significant cross attraction between different chemotypes and thus it can reasonably be concluded that pheromone communication (coupled with short-range stereotypic courtship behaviors or male courtship song) has contributed to divergence and potentially speciation in the *L. longipalpis* complex. The fact that males take multiple days to achieve maximum pheromone output suggests that there is a significant cost of pheromone production and recent transcriptomics and proteomics studies have revealed more about the biosynthetic pathway, but the precise evolutionary pathway across the *L. longipalpis* complex in unknown*.* Thus, we must assume that there are adaptive benefits of pheromone production including fitness benefits such as increased mating success and hence offspring production, contributing therefore to mate assessment. It remains a challenge to understand the evolutionary history of this group and the mechanisms underpinning its success in dispersion and adaptation.

## Abbreviations

1S: One spot phenotype
2S: Two spot phenotype
3MαH: (*1S,3S,7R*)-3-methyl-α-himachalene
9MGB: (*S*)-9-methylgermacrene-B
AVL: American visceral leishmaniasis
CEMB-1: Cembrene-1
CEMB-2: Cembrene-2
CHCs: Cuticular hydrocarbons
INS: Intermediate forms
SFPs: Seminal fluid proteins
